# Associations of Blood Pressure with the Factors among Adults in Jilin Province: A Cross-Sectional Study Using Quantile Regression Analysis

**DOI:** 10.1038/s41598-017-14045-0

**Published:** 2017-10-19

**Authors:** Junsen Ye, Zhongmin Li, Yaogai Lv, Lan An, Jianxing Yu, Xin Guo, Yan Yao, Yaqin Yu, Lina Jin

**Affiliations:** Epidemiology and Biostatistics, School of Public Health, NO. 1163 Xinmin Street, Jilin University, Changchun, Jilin, China 130021

## Abstract

Hypertension has become a major public health challenge. However, numerous research results reported in the literature focus primarily on risk factors of hypertension, little is known about how the whole continuum of blood pressure (BP) is associated with risk factors of hypertension. This study aims to reveal quantile-specific associations of BP with its risk factors. A cross-sectional survey based on a sample of 23,050 adults aged 18 to 79 years was conducted in Jilin Province in 2012, and some subjects were excluded due to missing values in BP or having BP control according to the purpose of this study. Quantile regression (QR) was employed to investigate the associations between systolic/diastolic blood pressure (SBP/DBP) and the risk factors. The SBP and DBP in males presented statistically higher than females (*P* < 0.001). High-salt diet for males manifested a slightly increasing positive association with higher SBP only for high quantiles (≥70), but with a higher DBP for middle part of the quantiles (30~75), compared with bland diet. High-salt diet, drinking and high-density lipoprotein cholesterol (HDL-C) were positively associated with BP measures in males. And the coefficient of total cholesterol (TC) in QR increased with BP in females who used to live in town.

## Introduction

Hypertension is believed as a significant risk factor for cardiovascular disease (CVD), and has brought heavy economic burden to individuals, families, and society^1–4^. It is a great challenge for public health worldwide, because of its high prevalence, which accounts for nearly half of the cardiovascular morbidity and mortality in the world^5,6^. In 2010, people died from hypertension approximately 2 million in China, accounting for about 24.60% of total mortality^7^. Therefore, hypertension is an important public health problem that are supposed to be addressed urgently.

At present, hypertension has become a hot research spot^8,9^, where most of studies focused primarily on hypertension, including prevalence, influencing factors, and health problems in cross-sectional and cohort studies^10–13^. Sun *et al*.’s findings suggested that waist circumference (WC) was an independent predictor of hypertension incidence^10^, and Zhang *et al*.’s prospective study suggested that body mass index (BMI) dynamic gain may be related to incident hypertension for men of all ages and young and middle-aged women^13^. Generally, hypertension was viewed as a categorical variable in these studies, but the incidence of hypertension is a chronic and continuous process. In other words, literatures about risk factors of hypertension have been mounting, yet there is a little of publications on determinants of the normal parts of blood pressure (BP).

Fortunately, quantile regression (QR) is not limited to explain the prevalence risk of hypertension. What’s more, it could also be used to explain the risk of the BP at any point on its distribution. QR comprehensively shows heterogeneous changes in the dispersion of the relationship of risk factors with BP continuum across its distribution. In addition, there is no distributional assumptions about the error term in QR model, thus, it enjoys high flexibility for modeling data with heterogeneous conditional distributions^14^.

In this study, we aimed mainly to investigate how the whole continuum of BP was associated with commonly researched influencing factors of hypertension using QR model. Jilin Province is situated in the northeast of China (latitude 40°~46°, longitude 121°~131°), with a cold climate and different residential dietary pattern^1,15^. Therefore, we could develop more effective prevention strategies for different BP populations, so as to achieve the purpose of “precision prevention”.

## Results

### Descriptive characteristics of participants by gender

Table 1 shows the basic characteristics of the participants, where BMI, SBP, DBP, TG and FPG were all significantly higher in males than those in females (*P* < 0.05), but age, TC, LDL-C and HDL-C were just the opposite (*P* < 0.05). And the proportions of demographics (drinking, smoking, hypertension, dyslipidemia and diabetes, etc.) were significantly different between genders. As shown in Table 2, the distribution of BP and quantiles between males and females were different. Therefore, we separately identified the factors for males and females by using QR model.

**Table 1:** Descriptive characteristics of participants by gender [$\overline{X}$ ± *S*, n(%)].

Variable	Total (n = 16524)	Male (n = 7607)	Female (n = 8917)	*t/χ2*	*P* value
Age (year)	47.79 ± 13.16	47.02 ± 13.73	48.45 ± 12.63	0.6893	<0.001
BMI (kg/m^2^)	24.26 ± 3.66	24.34 ± 3.66	24.19 ± 3.66	2.555	0.011
SBP (mmHg)	131.33 ± 21.33	134.46 ± 19.74	128.67 ± 22.25	17.750	<0.001
DBP (mmHg)	79.99 ± 11.73	82.34 ± 11.71	77.99 ± 11.38	24.095	<0.001
TG (mmol/L)	1.96 ± 1.80	2.17 ± 2.09	1.79 ± 1.49	13.508	<0.001
TC (mmol/L)	4.90 ± 1.08	4.88 ± 1.06	4.92 ± 1.10	−2.154	0.031
LDL-C (mmol/L)	2.94 ± 0.89	2.89 ± 0.86	2.97 ± 0.91	−5.690	<0.001
HDL-C (mmol/L)	1.39 ± 0.39	1.35 ± 0.41	1.42 ± 0.36	−11.936	<0.001
FPG (mmol/L)	5.39 ± 1.66	5.53 ± 1.68	5.27 ± 1.64	9.936	<0.001
Education					
Compulsory	4985(30.2)	1696(22.3)	3289(36.9)	422.429	<0.001
High school	8981(54.4)	4539(59.7)	4442(49.8)		
Undergraduate	2558(15.5)	1372(18.0)	1186(13.3)		
Occupation					
Other *	4018(24.3)	1218(16.0)	2800(31.4)	621.136	<0.001
Manual labor	7914(47.9)	4302(56.6)	3612(40.5)		
Mental labor	4592(27.8)	2087(27.4)	2505(28.1)		
Residence					
Rural	8484(51.3)	3959(52.0)	4525(50.7)	2.770	0.050
Town	8040(48.7)	3648(48.0)	4392(49.3)		
Drinking					
No	11393(68.9)	3302(43.4)	8091(90.7)	4295.061	<0.001
Yes	5131(31.1)	4305(56.6)	826(9.3)		
Smoking					
No	11512(67.9)	3634(47.8)	7878(88.3)	3198.384	<0.001
Yes	5012(30.3)	3973(52.2)	1039((11.7)		
high - salt diet					
No	10242(62.0)	4229(55.6)	6013(67.4)	244.190	<0.001
Yes	6282(38.0)	3378(44.4)	2904(32.6)		
Family history of CVD					
No	8456(51.2)	4158(54.7)	4298(48.2)	68.564	<0.001
Yes	8068(48.8)	3449(45.3)	4619(51.8)		
Hypertension					
No	10368(62.7)	4479(58.9)	5589(66.0)	90.089	<0.001
Yes	6165(37.3)	3128(41.1)	3028(34.0)		
Diabetes					
No	9942(60.2)	4229(55.6)	5713(64.1)	123.028	<0.001
Yes	6582(39.8)	3378(44.4)	3204(35.9)		
Dyslipidemia					
No	14855(89.9)	6793(89.3)	8062(90.4)	5.477	0.019
Yes	1668(10.1)	813(10.7)	855(9.6)		

* “other” included unemployed and retired people.

**Table 2:** Quantiles of blood pressure (SBP & DBP) by gender.

Variable	Quantiles											
	10	20	30	40	50	60	70	75	80	85	90	95
Male												
SBP(mmHg)	113	118	122	126	130	135	140	144	149	153	160	173
DBP(mmHg)	68	72	75	78	80	84	87	89	91	94	97	102
Female												
SBP(mmHg)	104	110	114	119	124	129	136	140	145	152	159	171
DBP(mmHg)	64	68	71	74	77	79	82	84	86	89	92	98

Numbers in the Table are blood pressure at different quantiles.

### QR statistics between the BP measures in females

Tables 3~4 show QR coefficients and 95% confidence intervals of the influencing factors for BP in females. Age was positively associated with both SBP and DBP. Similarly, BMI, TG, TC and family history of CVD showed significantly positive associations with SBP/DBP across the entire conditional BP distribution. Females who used to live in town were positively associated with a higher DBP/SBP for most of the quantiles (≥10). Undergraduate females were negatively associated with SBP for most of the quantiles (≥20). Middle school females were negatively associated with SBP for part of the quantiles. Female manual labors and mental labors were positively associated with a higher DBP for part of the quantiles, compared with unemployed and retired females.

**Table 3:** Quantile regression coefficients [95% confidence intervals] of influencing factors for SBP in females.

Variables		Quantile										
		10	20	30	40	50	60	70	75	80	85	90
Age (year)		0.33* [0.29,0.36]	0.43* [0.39,0.46]	0.50* [0.47,0.53]	0.59* [0.55,0.62]	0.67* [0.63,0.70]	0.75* [0.72,0.79]	0.82* [0.78,0.86]	0.88* [0.84,0.93]	0.94* [0.89,0.98]	1.02* [0.96,1.08]	1.07* [1.01,1.15]
BMI (Kg/m^2^)		1.00* [0.90,1.09]	0.93* [0.84,1.05]	1.06* [0.96,1.16]	1.09* [0.99,1.23]	1.22* [1.11,1.31]	1.23* [1.12,1.35]	1.28* [1.15,1.42]	1.28* [1.13,1.44]	1.32* [1.15,1.51]	1.48* [1.24,1.67]	1.63* [1.41,1.85]
Residence	Rural	—	—	—	—	—	—	—	—	—	—	—
	Town	0.30 [−0.42,1.03]	0.79* [0.29,1.78]	1.57* [0.84,2.14]	1.69* [0.92,2.40]	1.76* [1.02,2.42]	2.12* [1.18,2.79]	3.22* [2.35,4.01]	3.40* [2.21,4.61]	3.15* [2.18,4.35]	3.16* [1.92,4.89]	4.22* [2.78,5.63]
Family history of CVD	No	—	—	—	—	—	—	—	—	—	—	—
	Yes	1.97* [1.34,2.64]	2.24* [1.54,2.87]	2.76* [2.12,3.37]	2.92* [2.25,3.55]	2.90* [2.25,3.56]	2.96* [2.33,3.70]	2.74* [1.86,3.54]	3.14* [2.02,4.08]	3.43* [2.53,4.36]	4.14* [2.98,5.34]	4.89* [3.22,5.94]
Education	Compulsory	—	—	—	—	—	—	—	—	—	—	—
	High school	−0.71 [−1.52,0.17]	−0.83 [−1.54,0.24]	−0.57 [−1.47,0.27]	−0.81* [−1.61,−0.03]	−1.06* [−1.76,−0.31]	−0.94* [−2.09,−0.12]	−0.90 [−2.09,0.2]	−0.75 [−2.31,0.46]	−1.21* [−2.31,−0.02]	−1.68 [−3.41,0.23]	−1.81 [−4.34,0.28]
	Undergraduate	−0.96* [−1.97,−0.15]	−1.90* [−3.02,−0.69]	−1.66* [−2.48,−0.61]	−1.99* [−3.29,−0.89]	−2.12* [−3.3,−1.01]	−2.04* [−3.17,−0.92]	−2.63* [−4.2,−1.36]	−3.06* [−4.82,−1.34]	−3.96* [−5.33,−1.89]	−5.39* [−6.92,−3.05]	−5.91* [−8.71,−3.26]
Blood lipid	TG	0.81* [0.62,1.07]	0.77* [0.36,1.01]	0.81* [0.64,1.01]	0.75* [0.47,1.01]	0.87* [0.44,1.18]	0.90* [0.61,1.16]	1.18* [0.66,1.61]	1.43* [0.81,1.99]	1.59* [1.13,2.08]	1.77* [1.13,2.37]	1.89* [1.00,2.27]
	TC	0.15 [−0.10,0.62]	0.53* [0.21,0.86]	0.54* [0.28,0.83]	0.56* [0.13,0.97]	0.69* [0.33,1.06]	0.89* [0.38,1.28]	1.12* [0.55,1.53]	0.91* [0.14,1.49]	0.79* [0.28,1.31]	0.38 [−0.22,1.02]	0.30 [−0.40,1.29]

Numbers in the Table are coefficient estimates from the quantile regression with 95% confidence intervals shown in brackets.

^*^ *P* < 0.05.

**Table 4:** Quantile regression coefficients [95% confidence intervals] of influencing factors for DBP in females.

Variables		Quantile										
		10	20	30	40	50	60	70	75	80	85	90
Age (year)		0.04* [0.02,0.07]	0.07* [0.06,0.09]	0.09* [0.07,0.11]	0.11* [0.09,0.12]	0.12* [0.1,0.14]	0.14* [0.11,0.17]	0.15* [0.13,0.18]	0.17* [0.14,0.20]	0.19* [0.16,0.22]	0.20* [0.17,0.23]	0.23* [0.18,0.26]
BMI (Kg/m^2^)		0.64* [0.54,0.73]	0.68* [0.63,0.73]	0.71* [0.65,0.76]	0.72* [0.66,0.78]	0.77* [0.73,0.83]	0.81* [0.74,0.87]	0.82* [0.74,0.89]	0.86* [0.75,0.93]	0.85* [0.77,0.93]	0.85* [0.77,0.94]	0.97* [0.83,1.04]
Residence	Rural	—	—	—	—	—	—	—	—	—	—	—
	Town	0.37 [−0.18,1.21]	0.62* [0.18,1.08]	0.78* [0.32,1.24]	0.99* [0.51,1.46]	1.09* [0.82,1.53]	1.05* [0.56,1.49]	0.94* [0.32,1.47]	0.93* [0.41,1.52]	1.29* [0.38,1.83]	0.90* [0.19,1.65]	1.04* [0.05,1.87]
Family history of CVD	No	—	—	—	—	—	—	—	—	—	—	—
	Yes	1.40* [0.82,1.91]	1.32* [0.95,1.67]	1.49* [1.14,1.90]	1.70* [1.29,2.10]	1.51* [1.18,1.85]	1.84* [1.45,2.28]	2.24* [1.72,2.78]	2.39* [1.90,2.94]	2.68* [2.07,3.24]	2.78* [2.08,3.41]	2.64* [1.91,3.31]
Occupation	Other *	—	—	—	—	—	—	—	—	—	—	—
	Manual labor	0.59 [−0.30,1.46]	0.75* [0.27,1.20]	0.63* [0.05,1.20]	0.6* [0.09,1.25]	0.68* [0.16,1.06]	0.68* [0.13,1.23]	1.00* [0.33,1.65]	1.19* [0.60,1.92]	1.18* [0.58,2.13]	1.32* [0.60,2.09]	1.37* [0.24,2.43]
	Mental labor	0.09 [−0.95,0.96]	0.42 [−0.08,0.97]	0.46 [0.02,1.12]	0.36 [−0.15,1.01]	0.54 [−0.07,1.12]	0.70* [0.00,1.28]	0.99* [0.18,1.61]	0.99* [0.42,1.83]	1.20* [0.32,2.04]	0.73 [−0.25,1.58]	0.50 [−0.88,1.42]
Blood lipid	TG	0.35* [0.22,0.53]	0.40* [0.24,0.61]	0.62* [0.42,0.87]	0.71* [0.52,0.82]	0.62* [0.46,0.76]	0.67* [0.4,0.89]	0.72* [0.48,0.99]	0.75* [0.54,1.00]	0.80* [0.45,1.03]	0.68* [0.47,1.14]	0.78* [0.48,1.10]
	TC	0.72* [0.34,0.96]	0.57* [0.32,0.78]	0.47* [0.27,0.70]	0.53* [0.36,0.73]	0.60* [0.43,0.79]	0.51* [0.26,0.75]	0.59* [0.36,0.94]	0.69* [0.42,1.03]	0.72* [0.33,1.05]	0.86* [0.42,1.28]	0.73* [0.26,1.29]

Numbers in the Table are coefficient estimates from the quantile regression with 95% confidence intervals shown in brackets.

^*^ *P* < 0.05, “other” included unemployed and retired people.

### QR statistics between the BP measures in males

Tables 5~6 provide QR coefficients and 95% confidence intervals of the influencing factors for BP in males. Similar to females, age, BMI, TG and family history of CVD presented positive associations with both BP measures across the entire conditional BP distribution. Drinking showed positive associations with SBP/DBP. High-salt diet manifested a slightly increasing positive association with higher SBP (quantiles ≥ 70), but with a higher DBP for middle part of the quantiles (30~75), compared with the bland diet. Undergraduate males were negatively associated with SBP for most of the quantiles (≥20). Male manual labors and mental labors were positively associated with a higher DBP across the entire conditional DBP distribution compared with unemployed and retired males.

**Table 5:** Quantile regression coefficients [95% confidence intervals] of influencing factors for SBP in males.

Variables		Quantile										
		10	20	30	40	50	60	70	75	80	85	90
Age (year)		0.15* [0.11,0.19]	0.23* [0.19,0.26]	0.27* [0.24,0.31]	0.35* [0.31,0.38]	0.43* [0.39,0.46]	0.51* [0.46,0.54]	0.58* [0.54,0.60]	0.61* [0.57,0.65]	0.68* [0.64,0.73]	0.73* [0.68,0.79]	0.82* [0.76,0.88]
BMI (Kg/m^2^)		1.15* [1.00,1.26]	1.14* [1.04,1.26]	1.27* [1.18,1.40]	1.32* [1.23,1.43]	1.36* [1.25,1.46]	1.43* [1.28,1.52]	1.44* [1.30,1.55]	1.51* [1.36,1.63]	1.53* [1.35,1.73]	1.55* [1.37,1.76]	1.66* [1.46,1.90]
Drinking	No	—	—	—	—	—	—	—	—	—	—	—
	Yes	2.94* [2.19,3.79]	3.07* [2.30,3.81]	3.10* [2.48,3.79]	2.98* [2.32,3.7]	3.36* [2.75,4.17]	3.7* [2.74,4.50]	3.57* [2.51,4.34]	3.57* [2.66,4.49]	3.74* [2.33,4.81]	3.75* [2.50,5.17]	4.87* [3.12,6.16]
High - salt diet	No	—	—	—	—	—	—	—	—	—	—	—
	Yes	0.22 [−0.65,1.09]	0.24 [−0.49,0.90]	0.36 [−0.32,1.09]	0.15 [−0.49,0.88]	0.72 [−0.07,1.44]	0.74 [−0.13,1.68]	0.53 [−0.13,1.63]	1.10* [0.23,1.95]	1.47* [0.36,2.69]	1.45* [0.19,2.74]	2.35* [0.78,3.97]
Family history of CVD	No	—	—	—	—	—	—	—	—	—	—	—
	Yes	1.20* [0.42,2.06]	0.97* [0.18,1.65]	1.23* [0.60,1.91]	1.64* [0.92,2.32]	2.08* [1.25,2.78]	2.48* [1.50,3.31]	2.93* [2.01,3.77]	2.92* [2.02,3.68]	3.12* [1.96,4.21]	3.12* [1.58,4.13]	3.69* [1.99,5.21]
Education	Compulsory	—	—	—	—	—	—	—	—	—	—	—
	High school	0.03 [−0.92,1.01]	0.49 [−0.35,1.63]	0.39 [−0.73,1.31]	0.35 [−0.72,1.22]	0.85 [−0.30,1.66]	0.55 [−0.65,1.58]	0.87 [−0.43,1.98]	0.59 [−0.62,1.78]	0.22 [−1.10,2.03]	0.35 [−1.48,2.34]	−2.28 [−3.77,0.94]
	Undergraduate	−1.11 [−2.36,0.14]	−0.57 [−1.37,0.78]	−1.28* [−2.38, −0.09]	−1.27* [−2.60, −0.04]	−1.06* [−2.56, −0.08]	−1.43* [−2.93, −0.43]	−1.93* [−3.47, −0.53]	−2.26* [−3.46, −0.74]	−2.88* [−4.38, −0.71]	−2.63* [−5.11, −0.32]	−5.26* [−7.39, −2.68]
Blood lipid	TG	0.40* [0.27,0.69]	0.60* [0.34,0.81]	0.72* [0.51,0.97]	0.79* [0.55,0.94]	0.78* [0.58,1.07]	0.94* [0.61,1.14]	0.96* [0.73,1.28]	1.04* [0.72,1.33]	1.24* [0.78,1.72]	1.24* [0.68,1.74]	1.15* [0.75,1.87]
	HDL-C	2.58* [0.90,3.98]	3.04* [1.93,3.99]	3.63* [2.41,4.62]	4.00* [2.88,4.95]	3.63* [2.40,4.63]	3.96* [2.76,5.15]	3.63* [2.55,4.98]	3.78* [2.41,4.79]	3.97* [1.68,5.93]	4.25* [2.57,6.41]	4.69* [2.45,7.97]

Numbers in the Table are coefficient estimates from the quantile regression with 95% confidence intervals shown in brackets.

^*^ *P* < 0.05.

**Table 6:** Quantile regression coefficients [95% confidence intervals] of influencing factors for DBP in males.

Variables		Quantile										
		10	20	30	40	50	60	70	75	80	85	90
Age (year)		0.07* [0.04,0.09]	0.10* [0.07,0.12]	0.12* [0.10,0.14]	0.15* [0.13,0.17]	0.16* [0.14,0.18]	0.18* [0.16,0.21]	0.20* [0.18,0.23]	0.23* [0.20,0.25]	0.22* [0.20,0.27]	0.25* [0.22,0.29]	0.27* [0.22,0.31]
BMI (Kg/m^2^)		0.67* [0.57,0.76]	0.68* [0.60,0.75]	0.78* [0.70,0.86]	0.85* [0.78,0.92]	0.88* [0.82,0.96]	0.91* [0.84,0.98]	0.92* [0.86,1.02]	0.91* [0.83,1.02]	0.95* [0.84,1.03]	0.98* [0.87,1.08]	1.03* [0.87,1.17]
Drinking	No	—	—	—	—	—	—	—	—	—	—	—
	Yes	2.29* [1.69,2.85]	2.1* [1.47,2.59]	2.15* [1.74,2.72]	2.34* [1.91,2.77]	2.57* [2.01,3.03]	2.90* [2.44,3.42]	3.11* [2.47,3.75]	2.94* [2.34,3.67]	2.84* [2.22,3.57]	3.16* [2.22,3.89]	2.72* [1.84,3.41]
High - salt diet	No	—	—	—	—	—	—	—	—	—	—	—
	Yes	0.80* [0.19,1.38]	0.41 [−0.04,1.00]	0.71* [0.23,1.25]	0.82* [0.38,1.22]	0.80* [0.27,1.20]	0.87* [0.43,1.36]	0.89* [0.35,1.43]	0.83* [0.19,1.39]	0.70* [0.06,1.32]	0.56 [−0.05,1.43]	0.72 [−0.10,1.86]
Family history of CVD	No	—	—	—	—	—	—	—	—	—	—	—
	Yes	0.58* [0.06,1.29]	0.97* [0.51,1.51]	1.16* [0.67,1.65]	1.37* [0.92,1.85]	1.76* [1.31,2.23]	2.04* [1.50,2.53]	2.30* [1.74,2.87]	2.68* [2.02,3.22]	2.63* [2.00,3.29]	2.52* [1.82,3.28]	2.81* [2.09,3.64]
Occupation	Other*	—	—	—	—	—	—	—	—	—	—	—
	Manual labor	1.77* [0.71,3.17]	1.51* [0.46,2.12]	1.40* [0.71,2.05]	1.54* [0.81,2.00]	1.32* [0.46,2.13]	1.39* [0.55,2.33]	1.06* [0.26,1.82]	1.39* [0.26,2.08]	1.22* [0.38,2.20]	1.36* [0.36,2.20]	1.91* [1.15,3.03]
	Mental labor	2.03* [1.32,3.36]	2.21* [1.05,3.04]	2.36* [1.56,3.00]	2.47* [1.59,3.02]	2.18* [1.24,3.02]	2.01* [1.15,2.95]	1.52* [0.73,2.36]	1.79* [0.55,2.57]	1.45* [0.63,2.58]	1.51* [0.46,2.48]	2.42* [1.31,3.92]
Blood lipid	TG	0.50* [0.43,0.70]	0.76* [0.54,0.87]	0.74* [0.61,0.83]	0.75* [0.66,0.89]	0.76* [0.65,0.93]	0.78* [0.64,0.96]	0.79* [0.61,1.02]	0.87* [0.60,1.05]	0.85* [0.62,1.10]	0.87* [0.67,1.29]	1.25* [0.77,1.71]
	HDL-C	0.92* [0.17,1.38]	1.85* [0.84,2.52]	2.41* [1.67,2.85]	2.26* [1.75,3.08]	2.65* [1.92,3.47]	2.78* [1.92,3.40]	2.57* [1.68,3.71]	2.63* [1.89,3.43]	2.97* [1.94,4.01]	3.00* [2.19,4.86]	4.37* [2.84,5.77]

Numbers in the Table are coefficient estimates from the quantile regression with 95% confidence intervals shown in brackets.

^*^ *P* < 0.05, “other” included unemployed and retired people.

**Figure 1:**
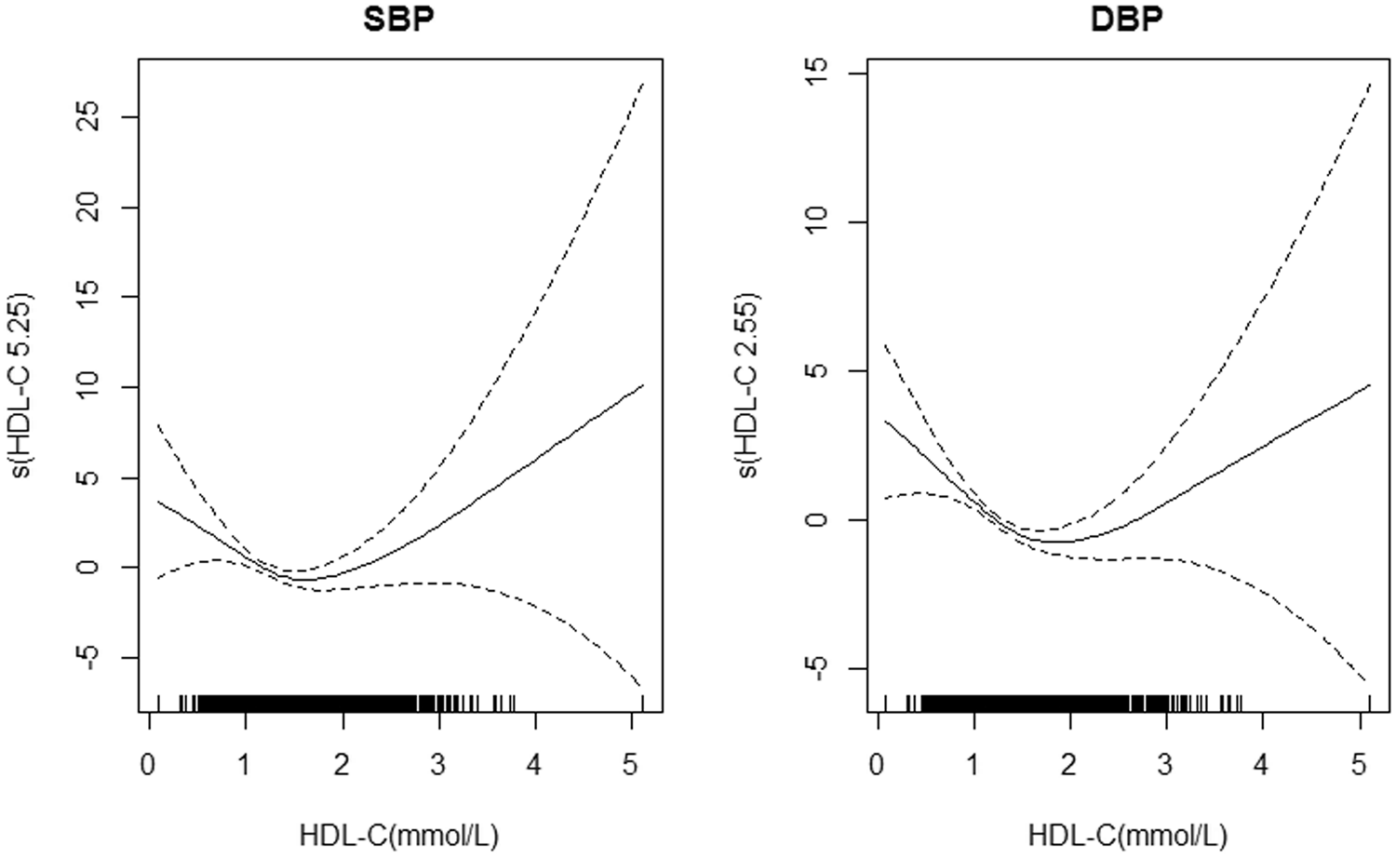
The association of BP and HDL-C in Generalized Additive Models (GAM). The vertical axis represents the smoothness function value, the numbers in brackets represent the estimated degrees of freedom (EDF), and the dotted line represents the lower limit of the confidence interval.

In addition, HDL-C also showed a positive association with SBP/DBP. In general, the quantiles of HDL-C among participants with hypertension were lower than those with normal BP in males (Table 7). In contrast, the relationship changed in different BMI groups: the relationship remained the same in normal weight group, however, the quantiles of HDL-C among participants with hypertension were higher than those with normal BP in underweight, overweight and obese groups. Further, the results of generalized additive models (GAM) showed that HDL-C performed non-linear associations with both SBP (*F* = 3.513, *P* = 0.012) and DBP (*F* = 7.388, *P* < 0.001) (Fig. 1).

**Table 7:** Quantiles of HDL-C for different BMI groups in males (mmol/L).

Quantiles	Total		Underweight		Normal		Overweight		Obese	
	Normal	Hypertension	Normal	Hypertension	Normal	Hypertension	Normal	Hypertension	Normal	Hypertension
10	0.92	0.88	1.01	0.98	1.11	1.13	0.87	0.88	0.80	0.82
20	1.04	0.99	1.14	1.13	1.30	1.29	0.97	0.98	0.90	0.90
30	1.13	1.09	1.23	1.24	1.47	1.36	1.05	1.06	0.97	0.98
40	1.22	1.17	1.32	1.33	1.67	1.46	1.11	1.15	1.03	1.04
50	1.30	1.26	1.40	1.44	1.78	1.53	1.19	1.22	1.09	1.11
60	1.39	1.35	1.49	1.55	1.90	1.63	1.26	1.31	1.15	1.18
70	1.50	1.46	1.61	1.68	1.97	1.72	1.35	1.41	1.22	1.25
75	1.57	1.54	1.68	1.75	2.13	1.79	1.40	1.46	1.26	1.30
80	1.64	1.62	1.75	1.85	2.23	1.92	1.47	1.53	1.30	1.36
85	1.74	1.72	1.85	1.96	2.33	2.03	1.54	1.60	1.35	1.43
90	1.87	1.86	1.98	2.13	2.58	2.26	1.67	1.71	1.42	1.50

Underweight: BMI < 18.5 kg/m^2^, Normal: 18.5 kg/m^2^ ≤ BMI < 24.0 kg/m^2^, Overweight: 24.0 kg/m^2^ ≤ BMI < 28.0 kg/m^2^, Obese: BMI ≥ 28.0 kg/m.

## Discussion

Hypertension has become a major public health problem in China^7^, and a number of researchers have investigated the risk factors of hypertension^5,16^, but little is known about how the whole continuum of DBP and SBP is associated with commonly researched influencing factors of hypertension. Measures in reducing the prevalence of hypertension in Jilin Province could be put forward after knowing the risk factors associated with BP. This study explored the heterogeneity of the relationship of environmental and individual determinants with hypertension across the entire conditional distribution of BP measures using a QR model.

It was indicated that unhealthy eating habits was a predictor for higher BP measures in males, with a larger extent of the positive association at a higher quantile. Previous studies have reported that BP was more prone to be elevated by alcohol drinking in males than in females^17^. In addition, it has been proven that high-salt diet was associated with BP^18^. Our results showed that the high-salt was positively associated with BP only for the males, and the mechanism was probably correlated with estrogen and salt sensitivity. Estrogen affects the sodium reabsorption and excretion via up regulation of NO production and different expression level of angiotensin receptors and ET receptors^19^. Therefore, males who maintain healthy lifestyle are also likely to manage their BP better, and thereby have a lower risk of hypertension.

Our study also investigated the effects of BMI and serum lipids on BP among adults. BMI showed a positive association with BP in our study, which was consistent with the literature that obese people were more likely to be hypertensive compared with nonobese people^20^. Activation of the renin–angiotensin system as well as physical compression of the kidney might be important factors in linking body weight and elevated BP^21^. Further, it has been proven that dyslipidemia was associated with BP^22^. Our results showed that TG level was positively correlated with BP, and the mechanism was probably related with the insulin resistance^23^. Elevated TG level could make a higher level of free fatty acids in serum, which would lead to insulin resistance, then insulin resistance further contributed to hyperinsulinemia, which might directly contribute to elevation of BP by increasing renal sodium retention^24^. However, TC and HDL-C showed different associations with BP between males and females, which might be attributed to differences in sex hormones, where estrogen was commonly believed to play an important role in lipolysis^25^. Moreover, HDL-C was positively associated with SBP/DBP in males, which was inconsistent with the results in literature^21,26^. One possible reason was the nonlinear relationship between HDL-C and SBP/DBP, which showed a “U” shape that both too lower and too higher levels of HDL-C might increase the risk of hypertension. Further, there might be an interaction between BMI and HDL-C, which showed that the HDL-C was higher among participants with hypertension than those with normal BP in almost all BMI groups except those with normal BMI. Besides, other confounding risk factors^27,28^ that were not under our consideration might also have impacts on this association.

Furthermore, it has been proven that education was associated with hypertension^29^. Our results showed that education negatively associated with SBP. On one hand, the people with higher level of education would have greater possibilities to engage in healthy lifestyles. On the other hand, older people were expected to have lower level of education than young people, which might also lead to higher SBP^30^. In addition, we also found that working people were more likely to have an elevated DBP, and the possible reason might be related to social engagement. Finally, the regression coefficient of family history of CVD increased with BP among all the research participates, which was consistent with other studies^31^. Thus, it was implied that elderly people with family history of CVD, low-literacy and workers should pay more attention to their BP levels.

Some limitations of our study should be noted. Firstly, the results were conducted from a cross-sectional study in Jilin province, which might limit our ability to generalize the results. Secondly, other confounders that might have implications for hypertension, such as parameters of glucose metabolism, renal function indices and genes, were not under our consideration this time, which might have some slight effects on our results.

## Conclusions

This study revealed interesting clues on how the whole continuum of SBP and DBP were associated with commonly researched factors of hypertension. High-salt diet, drinking and HDL-C were positively associated with BP measures only in males. And the risk of TC had an increased trend with BP in females who used to live in town. The elderly, the obese, workers and people with lower level of education and family history of CVD were expected to be positively associated with a higher SBP and/or DBP.

## Methods

### Study population

The large-scale cross-sectional survey was conducted among people who aged 18 to79 years old and were living in Jilin Province for over 6 months in 2012. A total of 23,050 participants were selected through multistage stratified random cluster sampling^32^ (see details in Part 1 of the Supplementary Material). For the purpose of the present analysis, the subjects who had anti-hypertensive treatments were excluded, as well as those who had missing values in SBP or DBP. Finally, a total of 16,524 subjects were included in the present analyses. All participants provided written informed consent, and the study was approved by the Institutional Review Board of the School of Public Health, Jilin University. And all methods were performed in accordance with the relevant guidelines and regulations.

### Data collection and measurement

The data of this study included demographics (e.g., gender, age, etc.), health-related behaviors (e.g., smoking, drinking, etc.), anthropometric measurements (e.g., height, weight, hypertension, etc.) and laboratory measurements (such as serum cholesterol and triglycerides). All investigation was trained and followed the same questionnaire instructions.

Height and weight were measured according to a standardized protocol and techniques, with the participants wearing clothing but no shoes. A calibrated mercury sphygmomanometer was used to determine the blood pressure of subjects on the right arm, after at least 5 min of seated rest. The blood sample was obtained in the morning from subjects after fasting for at least eight hours, and then conserved in tubes which contained ethylene diamine tetra acetic acid (EDTA). Fasting plasma glucose (FPG) and serum lipids were measured using a Bai Ankang fingertip blood glucose monitor (Bayer, Leverkusen, Germany) and a MODULE P800 biochemical analysis machine (Roche Co., Ltd., Shanghai, China), respectively^33^ (see details in Part 2 of the Supplementary Material).

### Assessment criteria

According to Seventh Joint National Commission Guidelines (JNC7), hypertension was defined as a resting systolic blood pressure (SBP)/diastolic blood pressure (DBP) ≥ 140/90 mmHg or current use of antihypertensive medication^34^. Dyslipidemia was defined as the use of lipid-lowering drugs in the past two weeks and/or meeting one or more of the following criteria: total cholesterol (TC) ≥ 6.22 mmol/L, triglyceride(TG) ≥ 2.26 mmol/L, low density lipoprotein cholesterol (LDL-C) ≥ 4.14 mmol/L, high density lipoprotein cholesterol (HDL-C) < 1.04 mmol/L^35^. We defined diabetes as participants who reported diabetes mellitus previously diagnosed by physician or those who have fasting plasma glucose (FPG) ≥ 7.0 mmol/L or oral glucose tolerance test (OGTT) 2 h plasma glucose (PG) ≥ 11.1 mmol/L^36^. BMI was defined as weight (kilogram) divided by height (meter) squared. Drinker was defined as a person who consumed more than one alcoholic drink per week, including any form of alcohol^37^. High-salt diet was defined as a person who daily salt intake more than 6 g^38^. Education stage was classified into compulsory education and lower education, secondary school education, senior school education, college education, postgraduate education and higher education.

### Statistical Analysis

All questionnaires were coded and double-entered. EpiData (version 3.1) was used for data entry and validation and R version 3.3.3(University of Auckland, Oakland, New Zealand) for data analysis. The data are presented as mean ± standard deviations (SD) or rate. In addition, quantitative variables and categorical variables were analyzed using *t* test and Rao-scott-*χ2* test, respectively. Finally, QR in the quantreg package was used to estimate the conditional quantile of the distribution of SBP and DBP under the influence of various risk factors.

### Data Availability

The survey was implemented by School of Public Health, Jilin University and Jilin Center for Disease Control and Prevention in Jilin Province in 2012. According to relevant regulations, we were sorry that the data can’t be shared.

### About the data

The survey was implemented by School of Public Health, Jilin University and Jilin Center for Disease Control and Prevention in Jilin Province in 2012. According to relevant regulations, we were sorry that the data can’t be shared.

## Electronic supplementary material

Supplementary information
