# Supplementary material for: Associations of Blood Pressure with the Factors among Adults in Jilin Province: A Cross-Sectional Study Using Quantile Regression Analysis
Source: Sci Rep. 2017 Oct 19;7:13613. doi: 10.1038/s41598-017-14045-0 (PMC5648827; doi:10.1038/s41598-017-14045-0)
Supplement: Supplementary file 1 — Supplementary information [file 41598_2017_14045_MOESM1_ESM.pdf]

Online Supplementary Material to

Associations of Blood Pressure with the Factors among Adults in Jilin Province: A Cross-Sectional Study Using Quantile Regression Analysis

Junsen Ye[a], Zhongmin Li[a], Yaogai Lv[a], Lan An[a], Jianxing Yu[a], Xin Guo[a], Yan Yao[a], Yaqin Yu[a], Lina Jin[a,*]

[a] Epidemiology and Biostatistics, School of Public Health, NO. 1163 Xinmin Street, Jilin University, Changchun, Jilin, China, 130021

* Correspondence: jinln@jlu.edu.cn; Tel.: +86-431-85619451;

1. Sampling Method

Five-stage stratified random cluster sampling was used to select the samples under study. In the first stage, 32 districts/counties were identified in proportion to population, geographic location and ethnicity, from nine cities (Changchun, Jilin, Siping, Liaoyuan, Tonghua, Baishan, Songyuan, Baicheng and Yanbian). At the second stage, three or four towns (depending on the size of the district) were selected by stratified random sampling to guarantee the representativeness of each sample. In the third stage, three neighborhood committees were chosen by stratified random sampling from each of the towns previously selected. In the fourth stage, one village from each chosen neighborhood committee was selected by simple random sampling. In the final stage, cluster random sampling was used to identify individuals aged 18 to 79 years old from each of the villages selected for the study.

23050 subjects were investigated in the study. For the purpose of the present analyses, some subjects were excluded due to the missing values and the other subjects were excluded due to the fact that they were limited to independent motor ability, daily life activity ability and walking ability, et al. Finally, a total of 16, 524 subjects were included in the present analyses.

2. Data Measurement

Anthropometric measurements including height, weight, blood pressure, serum lipids and fasting blood sugar were taken. During the interview, weight and height were determined though standardized protocol and measured in light indoor light clothing

without shoes. Weight was measured to the nearest 0.1 kg, and height, was measured to the nearest 0.1 cm.

A mercury sphygmomanometer was used to measure the blood pressure in the sitting position after a 10-min rest period. The appearance of the first sound was used to define systolic blood pressure (SBP) and the disappearance of sound was used to define diastolic blood pressure (DBP). Two readings each of SBP and DBP were recorded, and the average of each measurement was used for data analysis. If the first two measurements differed by more than 5 mmHg, additional readings were taken.

Blood samples were obtained from the antecubital vein into anticoagulant tubes containing EDTA in the morning after an overnight fasting period. All of the collected samples were transported on dry ice at prearranged intervals to the central laboratory. Serum lipids including TC, TG, HLD-C and LDL-C, which were measured by a MODULE P800 biochemical analyses machine.

Fasting plasma glucose (FPG) levels were measured using the Bayer Bai Ankang fingertip blood glucose monitor machine by taking a small drop of blood from a finger onto a strip of paper in the morning after participants fasted for 10 or more hours overnight.
